# Complete Genome Sequence of a Porcine Circovirus 2 Strain Isolated in Central China

**DOI:** 10.1128/genomeA.00268-14

**Published:** 2014-04-03

**Authors:** Guan-Peng Guo, Xin-Long Pan, Kun Cheng, Peng-Fei Fu, Ming-Fan Yang, Hong-Ying Chen

**Affiliations:** a College of Animal Science and Veterinary Medicine, Henan Agricultural University, Zhengzhou, Henan Province, People’s Republic of China; b Life Science College, Henan Agricultural University, Zhengzhou, Henan Province, People’s Republic of China

## Abstract

We report here the complete genome sequence of the porcine circovirus type 2 (PCV2) XZBK strain, isolated from central China. This is the first report of a member of the 1B subgroup of PCV2b central China. This finding will help in understanding the epidemiology and molecular characteristics of PCV2.

## GENOME ANNOUNCEMENT

Porcine circoviruses (PCVs) are circular single-stranded DNA viruses belonging to the genus *Circovirus* and the family *Circoviridae*, which contain porcine circovirus type 1 (PCV1) and type 2 (PCV2) (1). PCV1 does not induce disease in swine (2), while PCV2 is the etiological agent of postweaning multisystemic wasting syndrome (PMWS). This disease was first confirmed in Canada in 1997. Since then, it has brought great losses in pig production worldwide (3, 4). In 2001, PMWS associated with PCV2 infection in China occurred on many large pig farms in animals, and numerous PCV2 strains have been isolated since then (5–7).

PCV2 is one of the smallest DNA viruses infecting mammals, and its genome ranges from 1,766 to 1,768 nucleotides (nt). The PCV2 genome contains three major open reading frames (ORFs). ORF1 and ORF2 encode the replicase (Rep) protein and the viral capsid (Cap) protein, respectively, which perform two of the most elementary functions of the virus, copying and the successive packaging of the viral genome, and ORF3 encodes a protein that is involved in PCV2-induced apoptosis *in vitro*. PCV2 strains are divided into three PCV2 genotypes, PCV2a, PCV2b, and PCV2c (8). Recently, PCV2b has been the most prevalent strain in China (5, 7).

The PCV2 XZBK isolate was isolated from the intestine of a 4-week-old pig with naturally occurring diarrhea on a pig farm in Henan Province, central China. The whole genome of the PCV2 XZBK isolate was amplified by using the primers described by Fenaux et al. (9). The PCR products were purified and cloned into pMD18-T vector (TaKaRa Biotechnology, Dalian, China) and sequenced with an automated sequencer (3730xl Genetic Analyzer; Applied Biosystems). The result showed that the whole genome of the PCV2 XZBK strain is 1,767 nt in length, with a G+C content of 48.44%. XZBK contains at least three ORFs, encoding 2 major proteins (Rep and Cap). It has the sequence CCCCGC at nucleotides 262 to 267 of ORF2 and PR at amino acid positions 88 and 89 of ORF2. Thus, XZBK was classified in group 1. A comparison of genome sequences revealed that the PCV2 XZBK strain exhibited 95.8% to 99.3% nucleotide identity with other PCV2 strains in Henan Province and 95.6% to 99.2% with PCV2 genomes in other provinces in China. Phylogenetic analyses based on the genome of PCV2 strains indicated that XZBK together with PCV2b-1B (accession no. JF317566) from South Korea (10) was grouped in one cluster, and the genome of XZBK shared 99.7% nucleotide sequence identity to that of JF317566. There were only six nucleotide differences between the genomes of XZBK and JF317566. The nucleotide sequence and amino acid homologies between XZBK and PCV2b-1B (JF317566) were 99.6% and 100% in ORF1, respectively, while in ORF2, they shared 99.9% and 100% sequence homology at the nucleotide and amino acid levels, respectively. The results showed that the PCV2 XZBK strain belongs to the PCV2b-1B genotype.

### Nucleotide sequence accession number.

The genome sequence of the PCV2 XZBK strain has been deposited in GenBank under accession no. KF926650.

## References

[1] Finsterbusch T Mankertz A 2009 Porcine circoviruses—small but powerful. Virus Res. 143:177–183. 10.1016/j.virusres.2009.02.009 19647885

[2] Tischer I Bode L Peters D Pociuli S Germann B 1995 Distribution of antibodies to porcine circovirus in swine populations of different breeding farms. Arch. Virol. 140:737–743. 10.1007/BF01309961 7794114

[3] Mankertz A Domingo M Folch JM LeCann P Jestin A Segalés J Chmielewicz B Plana-Durán J Soike D 2000 Characterisation of PCV-2 isolates from Spain, Germany and France. Virus Res. 66:65–77. 10.1016/S0168-1702(99)00122-7 10653918

[4] An DJ Lim SI Kim YK Lee HK Cho YY Song JY Hyun BH Park BK 2014 Genetic characterization of porcine circovirus type 2 in the Korean wild boar population. Vet. Microbiol. 169:147–153. 10.1016/j.vetmic.2013.12.023 24480584

[5] Mu C Yang Q Zhang Y Zhou Y Zhang J Martin DP Xia P Cui B 2012 Genetic variation and phylogenetic analysis of porcine circovirus type 2 infections in central China. Virus Genes 45:463–473. 10.1007/s11262-012-0789-7 22843323

[6] Ge X Wang F Guo X Yang H 2012 Porcine circovirus type 2 and its associated diseases in China. Virus Res. 164:100–106. 10.1016/j.virusres.2011.10.005 22023739

[7] Li W Wang X Ma T Feng Z Li Y Jiang P 2010 Genetic analysis of porcine circovirus type 2 (PCV2) strains isolated between 2001 and 2009: genotype PCV2b predominate in postweaning multisystemic wasting syndrome occurrences in eastern China. Virus Genes 40:244–251. 10.1007/s11262-009-0438-y 20043196

[8] Olvera A Cortey M Segalés J 2007 Molecular evolution of porcine circovirus type 2 genomes: phylogeny and clonality. Virology 357:175–185. 10.1016/j.virol.2006.07.047 16963096

[9] Fenaux M Halbur PG Haqshenas G Royer R Thomas P Nawagitgul P Gill M Toth TE Meng XJ 2002 Cloned genomic DNA of type 2 porcine circovirus is infectious when injected directly into the liver and lymph nodes of pigs: characterization of clinical disease, virus distribution, and pathologic lesions. J. Virol. 76:541–551. 10.1128/JVI.76.2.541-551.2002 11752145 PMC136831

[10] Nguyen VG Kim HK Moon HJ Park SJ Keum HO Rho S Han JY Park BK 2012 Population dynamics and ORF3 gene evolution of porcine circovirus type 2 circulating in Korea. Arch. Virol. 157:799–810. 10.1007/s00705-012-1234-x 22289963

